# A study of negative life events driven depressive symptoms and academic engagement in Chinese college students

**DOI:** 10.1038/s41598-021-96768-9

**Published:** 2021-08-25

**Authors:** Lei Ji, Changfeng Chen, Binyin Hou, Decheng Ren, Fan Yuan, Liangjie Liu, Yan Bi, Zhenming Guo, Fengping Yang, Xi Wu, Xingwang Li, Chuanxin Liu, Zhen Zuo, Rong Zhang, Zhenghui Yi, Yifeng Xu, Lin He, Yi Shi, Tao Yu, Guang He

**Affiliations:** 1 Bio-X Institutes, Key Laboratory for the Genetics of Developmental and Neuropsychiatric Disorders, Shanghai Jiao Tong University, 1954 Huashan Road, Shanghai, 200030 China; 2 School of Mental Health, Jining Medical University, 16 Hehua Rd, Taibaihu New District, Jining, 272067 Shandong China; 3 Shanghai Key Laboratory of Psychotic Disorders, Brain Science and Technology Research Center, Shanghai Jiao Tong University, 1954 Huashan Road, Shanghai, 200030 China; 4 Shanghai Center for Women and Children’s Health, 339 Luding Rd, Shanghai, 200030 China

**Keywords:** Psychology, Risk factors

## Abstract

Negative life events (NLEs) are an important predictor of depressive symptoms (DS). College students experiencing NLEs are at risk of developing DS that could further weaken their academic engagement (AE), while social supports may assuage such negative effect. The aim of this study was to examine the relationship between negative life events, depressive symptoms, and academic engagement, and how the NLE-DS-AE relationship is affected by the level of social support among Chinese college students. To test this hypothesis, we applied data from the Decoding Happiness Gene Cohort Study (DHGCS). Baseline depressive symptoms and academic engagement were measured at the beginning of the first academic year. Approximately 12 months later, negative life events and social support over the past year were assessed retrospectively along with current depressive symptoms and academic engagement. A total of 3629 college students (Age = 18.67 ± 0.82) were included in the study. The prevalence of depressive symptoms was 26.7% and 36.7% in college students at the beginning of the first and second academic year, respectively. Depressive symptoms predicted subsequent academic engagement rather than the reverse based on cross-lagged analyses. Using structural equation modeling analyses, findings revealed a partial mediation effect of social support between negative life events and the development of depressive symptoms, and a partial mediation effect between negative life events and academic engagement. The findings presented negative life events jeopardize the academic engagement via depressive symptoms, while social supports are able to cancel such negative effect among college students under the Chinese cultural context.

## Introduction

Depressive symptoms (DS) are widely distributed among college students and this prevalence appears to increase over the last decade^1,2^. It showed that 33% college students have DS around the world, and the overall prevalence of depression was 23.8% among Chinese college students^3,4^. As a special group, college students endure a challenging stage, as it is accompanied by many abrupt and profound transitions in several aspects, such as being independent, social and academic life changes^5^. Notably, mild depressive symptoms could develop into a full-fledged depressive disorder without early intervention^6^, which can further trigger suicidal thoughts^7^; this holds true not only in general population, but also in clinical populations^7,8^. In addition to the physical damage, depressive symptoms can also impact the academic engagement and performance^9^.

DS might result from a series of events and processes, including genetic susceptibility, biological insults, individual characteristics, environmental events, and developmental changes. Studies have also shown that negative life events (NLEs) lead to increased risk of depression^10^. NLEs include events related to interpersonal relationship, academic stress, punishment, loss, health concerns, adaptation problem and other stress. A significant association between NLEs and the development of DS is relatively well established in Chinese college students^11^. However, it also has been recognized that many individuals who experience severe stressors do not develop DS. Potential protective factors (or buffer) in the development process of depression have been extensively studied, and social support has received particular attention^12^. To date, the predominant model has been the stress-buffering hypothesis, which posits that social support buffers the effect of stress on mental health^13^. According to the stress-buffering model, negative life events are linked to increased depressive symptoms, particularly when a person has limited rather than substantial social support^14^. The role of a strong social support network may reduce DS by strengthening students’ internal resources for coping with NLEs. However, evidence for the stress buffering hypothesis remains controversial. Cohen et al. measured stress, social support and depression at 11 and 22-week interval among American college students, and little longitudinal evidence was found supporting the stress buffer hypothesis^15^. Praharso et al. also found that social identity loss rather than social support can better predict reduced wellbeing following a stressful life event^14^, which provided further non-supportive evidence for the stress buffering hypothesis.

Through recent literature, Gariépy et al. stated that the knowledge gaps remained because of the measurement heterogeneity of social support^16^. Cultural differences in how people utilize their social support networks also add the complexity. When bringing personal problems to the attention of others for their help, people in collectivistic cultures may ask for social support with relatively more caution than those in the individualistic cultures because they hold the beliefs that individuals should not burden their social networks^17^. Even within a same culture, different groups may adopt different strategies to deal with NLEs. Among Chinese college students, the extent to which DS caused by negative life events can be alleviated by social support still needs studies.

Another factor closely related to students’ DS is the level of academic engagement (AE). Previous studies have found that depressive symptoms and academic performance are closely related among the adolescents^18,19^ and college students^20,21^. DS predicts poor academic performance, which in turn predicts depression in young adulthood^22^. In particular, changes in DS will further affect the changes in academic engagement among adolescents according to Rogers’s findings^23^. Sudents' academic performance is closely related to academic engagement in college students^24^. Unfortunately, to the authors’ knowledge, the possible direct relationship between DS and academic engagement among college students has not been tested previously. The causal relationship between depressive symptoms and academic engagement remains unclear among college students, especially in Chinese population with different culture background. In order to find the supportive evidence of the causal relationship between DS and AE, we expand the research group to occupational groups (AE in students corresponds to work engagement in occupational groups). Innstrand^25^ and Imamura^26^ found work engagement was more likely to be the antecedent for symptoms of depression than the outcome, while Judge and Locke supported a reciprocal relationship based on the cognitive theory of depression^27^. These studies with inconsistent results were conducted in Western nations. Given the negative correlation between AE and NLEs^28^, we would examine the longitudinal relationship between DS and AE among our sample before establish the NLE–DS–AE relationship.

There is evidence for positive relationship between academic engagement and social support. Jayarathna^29^ found that social support play an important role in the level of academic engagement during the first academic year in the university in Sri Lanka. In the three aspects of social support, social support from family and friends has a significant impact on the undergraduates’ academic engagement level but not the social support from significant others. Jayarathna also speculated that social support may reduce the stress due to the significant change from high school to university. Ganotice^30^ also showed that students with higher levels of social support had higher academic engagement and achievement score. Moreover, Pan^31^ found that adverse life experiences moderate the correlation among perceived social support and academic engagement among reconnected youth (mean age = 16.50).

Taken together, we hypothesize that negative life events significantly affect academic engagement through depressive symptoms among Chinese college students, and the negative effect can be alleviated by social support. We also identified several factors affecting DS based on previous findings, in order to exclude the influence of confounding factors. Potential covariates included gender, residence, minority, and the education level of the parents based on previous literature^20,32–36^. Most of the past research targeted western nations. Therefore, we establish a prospective cohort in a China university, and the data we used were collected in September 2016 (baseline/t_1_) and September 2017 (follow-up/t_2_), with an interval of 12 months. We measured the scores of DS and AE at the two time points, and the levels of negative life events and social support experienced between the two checkpoints. In this study, we aimed to (1) estimating the prevalence of DS among Chinese college students at the beginning of the first and second academic year; (2) investigating the relationship between NLEs, DS and AE; (3) Finally, clarifying the mediating role of social support on the NLE-DS-AE association. The study can provide some new evidence and insights for the role of social support on mental health and academic performance of young adults, especially under the Chinese cultural context.

## Methods

### Participants

**Figure 1:**
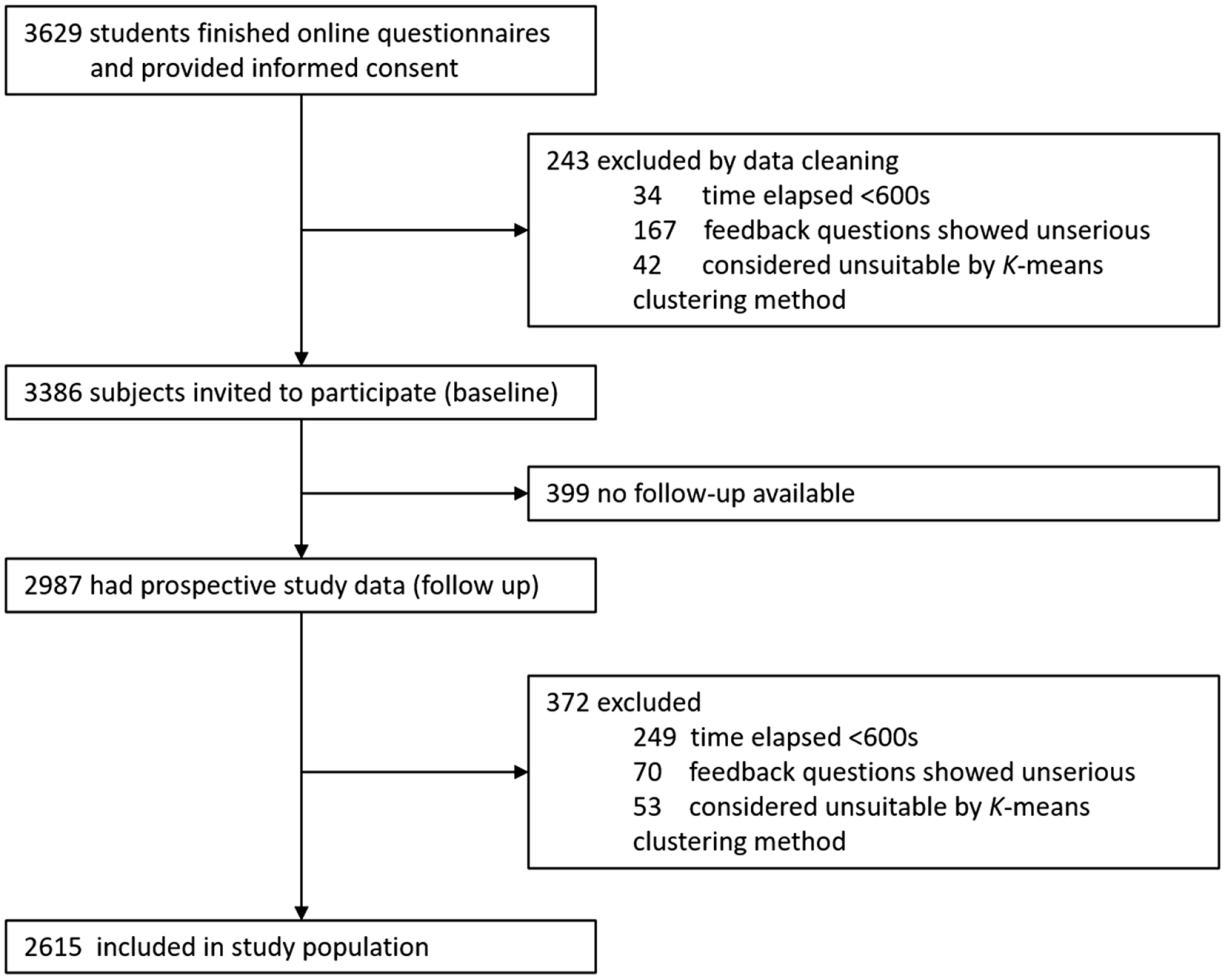
Trial profile.

This study is part of an ongoing school-based cohort study, the Decoding Happiness Gene Cohort Study (DHGCS), which aimed to investigate the longitudinal course of depressive symptoms and happiness as well as their affecting factors^37,38^. We recruited college students at the beginning of the first academic year and follow them up after 12 months, from a comprehensive university in Shandong, China (See supplementary Table S1 for details). In both study phases, we physically entered the campus to recruit the college students and gather them in the classrooms with the help of the school and teachers. In each classroom, a researcher would introduce the purpose and process of the study. Then, we uniformly distribute questionnaires through QR codes, and students fill in the questionnaires by scanning the codes on their mobile phones. At baseline, all 3629 freshmen were invited to take part in the cohort and fill in online self-report questionnaires, as well as feedback that reflects how carefully the subjects filled in (Fig. 1). Among them, 3386 (93.3%) responded with valid data. After the 12-month interval, 2987 (82.3%) could be reached and they were asked to assess negative life events and social support over the past 12 months, along with current levels of DS and AE. After exclusion, the final analysis included 2615 samples. In both baseline and follow up groups, exclusion criteria included elapse of questionnaire filling time < 600 s, feedback questions showed unserious (efficacy, understanding, carefulness, significance), and K-means clustering analysis filtering. The project was approved by the Ethics Committee of Jining Medical University and Shanghai human genetic resources ethics committee (JNMC-2016-KY-001), and all students had signed informed consents.

### Measures

#### Depressive symptoms (both waves)

Depressive symptoms (DSs) were assessed using the Chinese version of CES-D (Center for Epidemiologic Studies Depression Scale)^39,40^. The CES-D is a 20-item self-report measure of depression symptoms in the week preceding the screening. Each item is rated using a 4-point (0–3) Likert scale, and scores range from 0 to 60, with higher scores indicating a greater degree of depression symptoms. A cut-off score of ≥ 16 is a widely used indicator for likely clinically meaningful depressive symptoms^39,41^. The CES-D displayed good internal consistency (Cronbach's α = 0.87) and test–retest reliability (r = 0.65) in Chinese undergraduates^42^. The alpha coefficient in this study were 0.74 and 0.78 at baseline and follow-up, respectively.

#### Academic engagement (both waves)

The degree of academic engagement (AE) was measured by an update of the Chinese version of Utrecht Work Engagement Scale—Student (UWES-S)^43^. UWES-S is comprised of 17 items, and our scale has added 5 more on this basis (e.g., “I enjoy learning new things in class”), leading to a total of 22 items. The items are rated on a five-point Likert scale ranging from 1 (“Strongly disagree”) to 5 (“Strongly agree”). The total score is the sum of all items. In this study, the alpha consistency for this scale was 0.90 and 0.94 at baseline and follow-up, respectively.

#### Negative life events (follow-up)

Adolescent Self-Rating Life Events Check List (ASLEC) is used to evaluate the impact level of negative life events (NLEs) for adolescents in the past 12 months^44^. ASLEC is generally used to measure stress levels in Chinese young adults (mean age = 21.8 years)^45^ and college students^11^. ASLEC is composed of 26 items related to dimensions including interpersonal relationship, academic stress, punishment, loss, health and adaptation problem and other stress (e.g. ‘broken love’). The check list is a six-pointed scale (0 implies no negative life events, no impact; 5 implies negative life event experienced, serious impact). Cronbach’s alpha coefficient of the Chinese version of the scale was 0.92. The intensity of negative life events was calculated by summing up scores of all 26 items. Cronbach's alpha coefficient was 0.95 in our study.

#### Social support (follow-up)

We adopted the most widely used Social Support Scale for University Students (CAS-US) in Chinese college students to assess perceptions of their social support^46,47^. CAS-US is composed of 17 items including three dimensions: subjective support (SSP, reflects the level of satisfaction with the support received), objective support (OSP, reflects the amount of social support you can receive from others when you need it) and social support utilization (USP, reflects the extent to which individuals use social support). Each item is rated using a 5-point (1–5, strongly disagree—strongly agree) Likert scale. The higher total score implies the higher level of social support the participants receive. In the present sample, Cronbach’s α of the total items was 0.96. The final score of social support was calculated by confirmatory factor analyses (CFA) on these three dimensions. The result of the CFA provided an excellent fit to the observed data [χ^2^(3) = 426.475, p < 0.001]^48^. The loadings of the measured variables on the latent variable of social support were statistically significant at the 0.001 level, which implied that social support has been adequately measured.

### Covariates

A general questionnaire was designed to collect information on socio-demographics at baseline. Sociodemographic variables included were: gender (male or female), age, residence (rural or urban), only child (yes or no), minority (Han or other), parent education level (< high school or ≥ high school) and family income (< average or ≥ average). To identify potential covariates to include in subsequent analyses, t-tests and Pearson Correlations were conducted between sociodemographic variables and individual change of DS and AE scores (See supplementary Table S2 for details). Covariates were included in the model analyses if significant.

### Statistics analysis

All analyses were conducted in R studio version (4.0.2). Supplementary Table S3 displayed the R packages we used in this study. A p value < 0.05 was regarded as statistically significant. Mean ± SD (standard deviation) was used for the description of continuous variables and percentages for categorical data. T_1_ stands for baseline and t_2_ stands for follow-up. Depressive symptoms incidence rates for each time point were calculated by using 16 as a cut-off point. Pearson correlations tested relationships among variables using “Hmisc” and “corrgram” packages. To investigate the reciprocal relationship DS and AE have on each other, we conducted longitudinal cross-lagged models before path analysis. Cross-lagged analyses can test the causal structure, especially when the same variable is measured at two different times in the same sample^49^. For a comprehensive model on the direct and mediated paths from negative life events to the changes of DS and AE, we conducted path analysis (based on maximum likelihood estimations) using “lavaan” 0.6-7 package^50^. In all models, the covariates were controlled, and the p values presented in this article have been adjusted The indicators of model fit were: chi‐square values, root mean square error of approximation (RMSEA), comparative fit index (CFI), and Tucker–Lewis index (TLI)^51^. To assess the significance of the mediation effects, we used a recommended procedure and calculated the 95% confidence intervals of 1,000 bias‐corrected and accelerated bootstrapping analyses^52,53^. In order to chart change trajectories of DS and AE, we controlled for t_1_ measurements when predicting t_2_ measurements. Thus, t_2,1_ was interpreted as the change from t_1_ to t_2_.

### Ethics approval

The project has been approved by the Ethics Committee of the Bio-X Institutes in Shanghai Jiao Tong University. All procedures performed in studies involving human participants were in accordance with the ethical standards of the institutional and/or national research committee and with the 1964 Helsinki declaration and its later amendments or comparable ethical standards. All participants (2.2% subjects are under 18, informed consent was obtained from a parent and/or legal guardian) have signed informed consents form, agreed to participate in the project and the data provided can be used for publication.

## Results

### Preliminary analysis

Table 1 summarizes the descriptive statistics of the sample (*N* = 2615). The mean age was 18.82 ± 0.85 years at baseline. Female subjects (67.0%), rural residents (65.2%), Han nationality (97.1%) and people with parents' education level lower than high school accounted for the majority proportion among Chinese college population in this study. Supplementary Table S2 shows the result of analyses testing potential covariates. Female (p = 0.009), living in urban areas (p = 0.040), and mothers’ high education levels (p = 0.029) are risk factors for increased DS. According to these results, gender, residence, and the education level of mother were included as covariates in all analyses.

**Table 1:** Sample characteristics of the study population (baseline; *N* = 2615).

Characteristics	Mean	SD
Age	18.82	0.85
	N	%
^a^Gender: female	1752	67.0
^a^Residence: urban	910	34.8
Child: single	927	35.4
Minority: Han	2538	97.1
Family history of psychosis: yes	123	4.7
Family income: ≥ average	2085	79.7
Father education level: ≥ High school	1175	44.9
^a^Mother education level: ≥ High school	891	34.1

^a^There was significantly difference between factors and individual change of DS score using linear regressions (p = 0.009). Thus, gender, residence and the education level of mother would be used as the covariates in subsequent analysis.

Table 2 shows the mean scores and standard deviations of the variables, as well as the correlation matrix among all the variables included in the model. The overall average DS score was 11.75 ± 7.85 and 13.64 ± 8.47 at baseline and follow-up, respectively. As we used 16 as a cutoff point to indicate for clinically meaningful depressive symptoms, the incidence of DS was 26.7% at baseline and 36.7% at follow-up. Pearson correlations results showed that DS were positively and significantly correlated with NLE, whereas it was negatively associated with social support and AE. Social support and AE were positively associated with each other, and negatively related to NLE.

**Table 2:** Correlation matrix of variables (*N* = 2615).

	Mean (SD)	1	2	3	4	5	6	7	8
1 DS t_1_	11.75 (7.85)	1							
2 AE t_1_	80.39 (9.66)	− 0.4***	1						
3 DS t_2_	13.64 (8.47)	0.52***	− 0.24***	1					
4 AE t_2_	77.58 (11.38)	− 0.26***	0.47***	− 0.39***	1				
5 SSP	19.95 (3.96)	− 0.33***	0.27***	− 0.47***	0.37***	1			
6 OSP	25.07 (4.48)	− 0.28***	0.22***	–0.44***	0.33***	0.74***	1		
7 USP	23.52 (5.02)	− 0.26***	0.22***	− 0.39***	0.33***	0.76***	0.75***	1	
8 NLE	18.91 (16.75)	0.29***	–0.13***	0.44***	− 0.21***	− 0.32***	− 0.32***	–0.26***	1

*DS* depressive symptoms, *AE* academic engagement, *SSP* subjective social support, *OSP* objective social support, *USP* utilization of social support, *NLE* negative life events.

### Cross-lagged analysis

**Figure 2:**
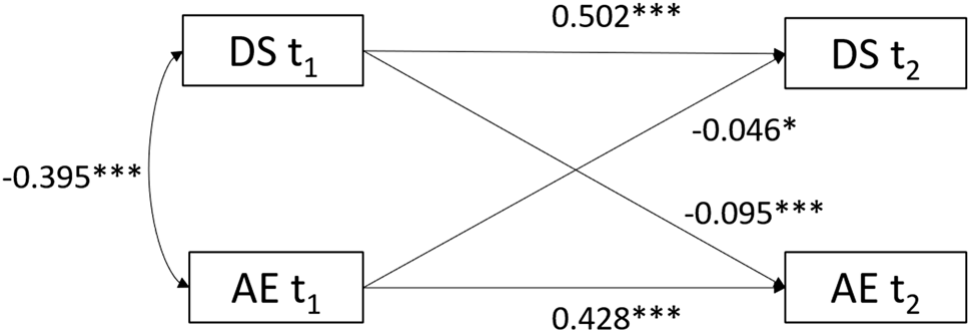
Cross-lagged path analysis of depressive symptoms and academic engagement. All estimates are standardized, with* indicating p < 0.05,** indicating p < 0.01,*** indicating p < 0.001.

Figure 2 showed the result from the cross-lagged analyses after controlling for covariates. The results showed, first, that students’ DS and AE showed moderate stability across the two measurements. Second, DS and AE were also negatively associated at each measurement. Third, DS at baseline predicted AE at follow-up (β =  −0.09; p < 0.001). Similarly, the reversed negative effect was supported since AE at baseline had a negative cross-lagged effect on DS (β =  −0.05; p = 0.017). Although the magnitude of the effects was both relatively weak, the effects of DS on AE were stronger than AE on DS. Thus, we used the DS score as the cause in the subsequent structural equation model.

### Path analysis of the mediation model

**Figure 3:**
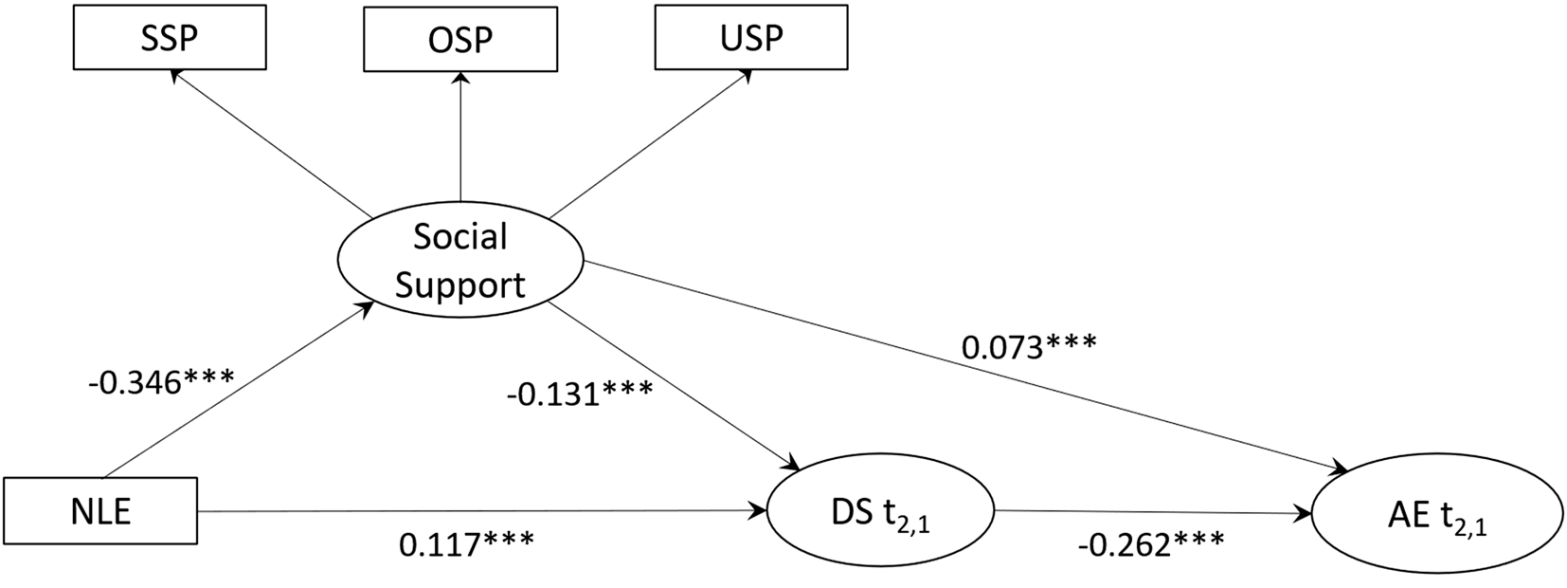
The mediation model of negative life events on depressive symptoms. Path analysis linking negative life events, social support with the change of depressive symptoms and academic engagement, adjusted for covariates (covariates included gender, residence and education level of mother). *Note*: t2,1 stands for measurement at follow-up while controlling for baseline. All estimates are standardized, with* indicating p < 0.05,** indicating p < 0.01,*** indicating p < 0.001. The overall model showed an adequate fit to the data: χ^2^(19) = 132.79, p < 0.001, RMSEA = 0.048 with lower 90% CI = 0.040 and higher 90% CI = 0.056, CFI = 0.980, TLI = 0.968.

The direct path coefficient from the predictor (NLEs) to the DS and AE in the absence of the mediators was significant (β = 0.156; p < 0.001), indicating that NLEs were positively associated with DS and subsequently resulted in a decrease in AE. The mediation model and a direct path from NLEs to DS and AE were established and was shown in Fig. 3. After controlling for covariates, the final model showed a good model fit. Specifically, NLEs were negatively associated with social support (β =  −0.346, p < 0.001) and positively associated with DS (β = 0.117, p < 0.001). On the contrary, social support was negatively correlated to DS (β =  −0.131, p < 0.001) and positively correlated to AE (β = 0.073, p < 0.001). The results indicated that college students experiencing higher levels of negative life events were more likely to report lower levels of social support, and their depressive symptoms might increase and academic engagement might decrease.

### Indirect effects

The results from bootstrapping showed that NLEs were indirectly related to DS via social support, with standard indirect effects of 0.045 (95% CI = [0.028, 0.062]). The indirect effects explained 27.78% of the variance in the changes of DS. Besides, NLEs were related to AE via social support and DS, with standard total effects of − 0.068 (95% CI = [− 0.086, − 0.049]). In this model, social support played a protective role in the effects of negative life events on depressive symptoms and academic engagement.

## Discussion

In this study, we first identified the causal relationship between depressive symptoms and academic engagement among Chinese college students. Two waves cross-lagged model suggested a negative correlation and depressive symptom was the antecedent variable and academic engagement were the outcome variable. By identifying the longitudinal effects of depressive symptoms, a new path was added in the structural equation model. We examined the association between negative life events and the individual changes of depressive symptoms, in addition to the mediating effect of social support on this association. Our findings supported that negative life events were directly associated with the increased depressive symptoms and decreased academic engagement, but such an association could be indirectly assuaged by social support. Identifying the risk and protective factors at the development of depressive symptoms is of key value for predicting changes in depressive symptoms, coping with the psychological impact on college students’ health, mitigating the increase in depressive symptoms, and preventing subsequent decline in academic engagement.

Based on our sample pool, the incidence of DS among Chinese college students was estimated to be 26.7% in the first academic year and 36.7% in the second academic year, which is higher than among college students in Beijing (24.8%) ≥ 16 but lower than among students in Hong Kong (43.9% ≥ 16)^54^. The prevalence of DS among total students increased in the second academic year, which indicates that the depression state of college students is not constant during the college period. Since this was a cohort project, no intervention has been taken at this time, but hopefully our study would provide help for school psychological intervention guidance. Our baseline values were measured within two weeks of freshmen enrollment, while follow-up was measured within two weeks at the beginning of the second academic year, when the student had experienced a year of university life. Due to this year's freshman life, college students are in a state of dysphoria, a new social network, and more trivial life events that are completely different from high school, which caused an increase in depression. This result is similar to Song’s investigation of depression in Chinese college students^55^.

First, we examined the cross-lagged paths between depressive symptoms and academic engagement among Chinese college students. To our best knowledge, this is the first study to study the causal relationship between depressive symptoms and academic engagement in the Chinese population. The result revealed that depressive symptoms predicted subsequent academic engagement rather than the reverse. College students with more severe depressive symptoms not only had less academic engagement in the current year, but also had a further impact on the academic engagement in the second year. The present result is inconsistent with previous investigations that found work engagement predicted symptoms of depression^21,22,25^. This may be due to the differences in the culture and educational philosophy of the students. Chinese parents’ expectations for their children’s academic performance are much stronger than those of western countries. Once a student has depressive symptoms, it is immediately reflected in their academic engagement. Depressive symptoms can affect one’s concentration, decision-making and action^5^. For students, it leads to low interest and motivation in learning, and then leads to a decrease in academic commitment. But we should point out that academic engagement is affected by many other factors, not just depressive symptoms. Specifically, the lagged negative effect of depressive symptoms on academic engagement 12 months later indicates that we must pay special attention to the psychological development of students.

We found a significant positive association between negative life events and the development of depressive symptoms after controlling for covariates. This suggests that college students experiencing higher levels of negative life events are more likely to have elevated depressive symptoms. Similar to our findings, Zou et al. found that the intensity of negative life events showed a strong positive correlation with depressive symptoms among male senior college students^11^. The subjects in their study were all male students, and the influence of gender could not be demonstrated. In addition, their study was a cross-sectional design, which might overlook a cause-and-effect relationship. O'Donnell’s finding of a positive relationship between NLEs and suicidal ideation is instructive, when comparing results with the studies conducted on the clinical populations^7^. In subsequent studies, NLEs and DS were measured close in time, and at multiple times during the course of follow-up, allowing for assessment of recent stressors and subsequent depressive state. Meanwhile, we did not consider variables such as intelligence, personality, entrance examination scores, and the selected majors as covariates. Since these factors may also affect the ability of college students to cope with negative life events, according to previous research. In order to make the study more reliable and persuasive however, we will consider these variables in future studies.

Given that negative life events are well established as a risk factor for depressive symptoms, we hypothesize that this relationship is mediated by social support. To determine the role of social support in the association between negative life events and the development of depressive symptoms among Chinese freshmen, the mediating effect of social support was tested. The finding in the present cohort of a negative correlation between negative life events and social support, and between increased depressive symptoms and social support, suggests that social support may alleviate the development of depressive symptoms associated with negative life events^12^. Besides, the findings also revealed a partial mediation effect of social support between negative life events and the changes in academic engagement. Our findings were inconsistent with Praharso et al.'s research, which indicated maintaining multiple group memberships, rather than one's degree of social support to the negative life events, is protective against a subsequent decline in wellbeing^14^. In our study, the effect of social support on depressive symptoms and academic engagement depends on its frequency, subjective experience and utilization efficiency, rather than the social support scale used in previous studies. Although the three dimensions were negatively associated with depressive symptoms, it is important to note that these 3 measures evaluate different constructs of social support and take more into consideration the social network and the social identity This result might be of significance for mental health policy making among college students in China.

A major strength of this study was the longitudinal testing of the hypotheses. This fills the gap in the literature by exploring the relationship between negative life events, depressive symptoms, and academic engagement, and further to examine how the NLE-DS-AE relationship is affected by the level of social support among Chinese college students. There are also several limitations in this work that needs future studies to address. The current sample was comprised of a medical university in Shandong province and spans only two years. The results apply only to an individual university or region. Therefore, generalizing the results to cover more diverse China college population will be done with caution in the future. Data collected in this study are also subject to measurement bias. For example, depressive symptoms were susceptible to the situation or emotional state at the time. We will plan to use an alternative method of empirical sampling method—Day Reconstruction Method (DRM) to reduce memory biases that are inherent in the recall of feelings^56^.

Our cohort is composed of young and developing students who, on average, have just entered or are still within the period of risk for depression. Continued follow-up will assist in investigating the risk of depression and evaluating the validity in other cohorts. Our cohort study is still ongoing, and more research results are expected in the future.

## Conclusion

Our findings add to the evidence and insights suggesting that social supports mediate the relationship between negative life events, the development of depressive symptoms, and the change of academic engagement among college students under the Chinese cultural context.

## Supplementary Information

Supplementary Tables.

## Data Availability

The data are not publicly available due to privacy or ethical restrictions.
