# Supplementary material for: A study of negative life events driven depressive symptoms and academic engagement in Chinese college students
Source: Sci Rep. 2021 Aug 25;11:17160. doi: 10.1038/s41598-021-96768-9 (PMC8387499; doi:10.1038/s41598-021-96768-9)
Supplement: Supplementary file 1 — Supplementary Tables. [file 41598_2021_96768_MOESM1_ESM.docx]

**Supplementary Table S1** Proportion of college students in each province

| Province | Count | Proportion |
| --- | --- | --- |
| Shandong Province | 2852 | 78.57% |
| Guizhou Province | 83 | 2.29% |
| HenNan Province | 71 | 1.96% |
| Anhui Province | 56 | 1.54% |
| Heilongjiang Province | 53 | 1.46% |
| Jilin Province | 42 | 1.16% |
| Jiangsu Province | 39 | 1.07% |
| Liaoning Province | 39 | 1.07% |
| Zhejiang Province | 38 | 1.05% |
| Hebei Province | 37 | 1.02% |
| Sichuan Province | 36 | 0.99% |
| Hubei Province | 31 | 0.85% |
| Jiangxi Province | 29 | 0.80% |
| Inner Mongolia Autonomous Region | 29 | 0.80% |
| Yunan Province | 28 | 0.77% |
| Gansu Province | 27 | 0.74% |
| Guangxi Zhuang Autonomous Region | 27 | 0.74% |
| Chongqing Province | 27 | 0.74% |
| Shanxi Province | 19 | 0.52% |
| Shanxi Province | 18 | 0.50% |
| Ningxia Hui Autonomous Region | 10 | 0.28% |
| Qinghai Province | 10 | 0.28% |
| Tianjin Province | 10 | 0.28% |
| Fujian Province | 6 | 0.17% |
| Guandong Province | 5 | 0.14% |
| Beijing Province | 4 | 0.11% |
| Shanghai Province | 4 | 0.11% |

**Supplementary Table S2** Potential Covariates and their Effects on the development of depressive symptoms and academic engagement

| **Characteristics** | Depressive symptoms t_2,1_ | |  | Academic egagement t_2,1_ | |
| --- | --- | --- | --- | --- | --- |
|  | **t** | **p** |  | **t** | **p** |
| Age | -1.374 | 0.170 |  | 0.530 | 0.596 |
| Gender: Female | 2.605 | **0.009** |  | 0.744 | 0.457 |
| Residence: Urban | 2.057 | **0.040** |  | -0.870 | 0.384 |
| Child: Single | 1.822 | 0.069 |  | -1.360 | 0.174 |
| Minority: Han | 0.898 | 0.369 |  | -1.461 | 0.144 |
| Family history of psychosis: Yes | -0.229 | 0.819 |  | -0.835 | 0.404 |
| Family income: ≥average | 0.076 | 0.940 |  | 0.004 | 0.997 |
| Father education level: ≥High school | 0.625 | 0.532 |  | -0.550 | 0.582 |
| Mother education level: ≥High school | 2.191 | **0.029** |  | -0.399 | 0.690 |

Note. We used simple linear regression to identify potential covariates that may influence the change of DS or AE scores: a single scalar predictor variable (sociodemographic) and a single scalar response variable (the change of DS or AE scores, respectively). The equation for this regression is represented by: y=a+bx.

**Supplementary Table S3** R packages in this study

| R package | version | URL | Description |
| --- | --- | --- | --- |
| Hmisc | 4.4-2 | https://hbiostat.org/R/Hmisc/ | Calculates correlation of variables |
| corrgram | 1.13 | https://github.com/kwstat/corrgram | Displays the results graphically. |
| lavaan | 0.6-7 | http://lavaan.org | Fit a variety of latent variable models, including confirmatory factor analysis, structural equation modeling and latent growth curve models. |
| semPlot | 1.1.2 | https://github.com/SachaEpskamp/semPlot | Path diagrams and visual analysis of various SEM packages' output. |
